# Residues of Tetracycline, Erythromycin, and Sulfonamides in Beef, Eggs, and Honey from Grocery Stores in Knoxville, Tennessee, USA: Failure of Cooking to Decrease Drug Concentrations

**DOI:** 10.3390/vetsci11120660

**Published:** 2024-12-17

**Authors:** Shamim Sarkar, Stephen A. Kania, Mohamed A. Abouelkhair, Brian Whitlock, Chika C. Okafor

**Affiliations:** 1 Department of Biomedical and Diagnostic Sciences, College of Veterinary Medicine, University of Tennessee, 2407 River Drive, Knoxville, TN 37996, USA; shamim.sarkar08@gmail.com (S.S.); skania@utk.edu (S.A.K.); abouelkhair@rowan.edu (M.A.A.); 2 Large Animal Clinical Sciences Department, College of Veterinary Medicine, University of Tennessee, 2407 River Drive, Knoxville, TN 37996, USA; bwhitloc@utk.edu

**Keywords:** antibiotic residues, antimicrobial residues, tetracycline, erythromycin, sulfonamide, antimicrobial resistance, beef, egg, honey, ELISA, conventional grocery store, Knoxville, East Tennessee

## Abstract

This study evaluated the concentrations of tetracycline, erythromycin, and sulfonamide in beef, eggs, and honey products sold at grocery stores and whether standard cooking to 160 °F (71 °C) reduced the concentrations of antimicrobials spiked into the evaluated food products. In June 2021, the concentrations of selected antimicrobials in 65 samples (8 beef, 48 eggs, 9 honey samples) purchased from grocery stores within Knoxville, Tennessee, were measured using enzyme-linked immunosorbent assays. The Kruskal–Wallis test was used to assess the differences in the median tetracycline concentrations across food types, while the Mann–Whitney test was used to compare the erythromycin residue concentrations between beef and honey. Linear regression was used to analyze whether standard cooking at 160 °F (71 °C) reduced antimicrobial concentrations. Detectable tetracycline concentrations were found in all beef (8/8, 100%), eggs (46/48, 96%), and honey (9/9, 100%) samples, with median concentrations of 7.73, 5.62, and 13.13 µg/kg, respectively. Honey had significantly higher tetracycline concentrations than eggs (*p* = 0.002). Detectable erythromycin concentrations were found in beef (5/8, 63%) and honey (9/9, 100%), with median concentrations of 0.14 µg/kg for beef and 0.48 µg/kg for honey. None of the antimicrobial concentrations found in the beef and egg products exceeded the U.S. FDA’s maximum residue limits (MRL). Sulfonamide concentrations were undetectable in the beef and egg products. The concentrations of tetracycline, erythromycin, and sulfonamide spiked into the food products did not change significantly in response to cooking to 160 °F (71 °C). The antimicrobial concentrations found in the selected commercial food products were below the MRL, and cooking appeared to not reduce the spiked antimicrobial concentrations in the evaluated food products.

## Simple Summary

This study assessed the concentrations of tetracycline, erythromycin, and sulfonamide in beef, eggs, and honey products sold at grocery stores in Knoxville, Tennessee, and whether the standard cooking temperature reduced the concentrations of antimicrobials spiked into the evaluated food products. Antimicrobial concentrations were measured using enzyme-linked immunosorbent assays (ELISAs). All tested food products had detectable tetracycline concentrations, with honey showing significantly higher concentrations than eggs. While erythromycin concentrations were detected in the beef and honey products, none of the amounts in the beef products exceeded the U.S. Food and Drug Administration (FDA) maximum residue limits (MRL). Sulfonamide concentrations were undetectable in the beef and egg products. The concentrations of tetracycline, erythromycin, and sulfonamide spiked into the food products did not change significantly in response to a cooking temperature of 160 °F (71 °C). Further research is needed to identify the sources of these antimicrobial concentrations and to develop strategies to mitigate their presence in the food supply.

## 1. Introduction

Animals that are raised for food often receive antimicrobials to treat, prevent, and manage bacteria-associated illness [1,2]. The use of antimicrobials in animal husbandry is common worldwide, with almost 80% of global antimicrobial production being used for animals [3,4]. This widespread usage has led to the presence of antimicrobial concentrations in food products derived from animals, such as meat, milk, eggs, and honey [5]. Regulations and monitoring systems for antimicrobial use vary widely worldwide. The European Union has implemented strict controls, including a ban on antimicrobials for growth promotion. In contrast, regions like parts of Asia and Africa often have less stringent monitoring and enforcement mechanisms [6].

Tetracycline and sulfonamides are the most commonly used antimicrobials in food-producing animals in the United States (U.S.) [7]. In 2018, the U.S. Food and Drug Administration (FDA) reported that 4389 tons of tetracycline, 334.5 tons of sulfonamides, and 532 tons of macrolides were sold for use in food-producing animals in the U.S. [7]. The presence of high concentrations (exceeding safe concentrations) of antimicrobials in food produced from animals is linked to the misuse of antimicrobials, the failure to follow antimicrobial withdrawal times, and the contamination of the environment with antimicrobials [8,9]. Long-term exposure to even small amounts of antimicrobial concentrations in food can have negative health consequences for consumers and increase the risk of the development of antimicrobial resistance (AMR) bacteria [10,11]. These antimicrobial concentrations may result from the misuse or overuse of antimicrobials in animal husbandry and agriculture [12]. Antimicrobial residues in food products can create selective pressure that fosters the emergence and spread of AMR bacteria. These resistant bacteria can transfer resistance genes to other microbes through mechanisms like horizontal gene transfer, thereby facilitating the dissemination of resistance traits within microbial communities [12,13,14].

AMR bacterial infections are a major public health threat in the U.S., with 2.8 million cases reported each year, according to the U.S. Centers for Disease Control and Prevention (CDC). In January 2017, the U.S. FDA implemented changes to the veterinary feed directive (VFD) final rule to restrict the use of medically important antimicrobials in animal feed, except to treat illnesses, and to slow AMR development [15].

Antimicrobial residues in animal-derived foods are a global concern, with evidence of antimicrobial residues in meat, eggs, and honey sold at retail grocery stores in Asia [16], Africa [17,18], and Europe [19,20]. The surveillance of antimicrobial residues in foods of animal origin is crucial to protect the health and welfare of consumers in the U.S. [21,22]. Residues of tetracycline and sulfonamide have been detected in meat and poultry products at slaughtering establishments across the U.S. [21]. Nonviolative concentrations of tetracycline, erythromycin, and sulfonamide were detected in “antibiotic-free” animal products sold at farmers’ markets in Knoxville, Tennessee [23]. The enzyme-linked immunosorbent assay (ELISA) has become a valuable method for detecting antimicrobial residues due to its high-throughput screening capability, versatility, and excellent specificity and sensitivity [24]. These features make ELISA an essential tool for screening for antimicrobial residues in food safety assessments.

The concentrations of antimicrobials in food animal products can be reduced by cooking food thoroughly. However, studies show that cooking temperature may have varying effects on the tetracycline and sulfonamide concentrations in food, and the results are inconsistent [25,26,27,28,29,30,31]. For example, a study reported that cooking reduced the tetracycline residues in chicken meat [28]. On the other hand, cooking was found to increase tetracycline and sulfonamide concentrations in ground beef [32]. Yet, no investigations have explored the impact of the USDA’s recommended cooking temperature, for inactivating most pathogenic bacteria, on the concentrations of antimicrobials spiked into food products.

This study evaluated the concentrations of tetracycline, erythromycin, and sulfonamide in beef, eggs, and honey products sold at grocery stores in Knoxville, Tennessee, and explored whether the standard cooking of food products to 160 °F (71 °C) reduced the concentrations of the antimicrobials spiked into the evaluated food products. The results provide quantitative data on the concentrations of tetracycline, erythromycin, and sulfonamide in food products sold at grocery stores and on the influence of cooking temperature on the concentrations of antimicrobials in food products. Assessing the antimicrobial concentrations in food products sold at grocery stores as well as examining how cooking temperature influences the concentrations of these antimicrobials in food products, are crucial for addressing potential food safety concerns and safeguarding consumer health.

## 2. Materials and Methods

### 2.1. Study Design, Area, and Sample Collection

In June 2021, a cross-sectional study was conducted to screen the beef, eggs, and honey sold at conventional grocery stores in Knoxville, TN, for concentrations of tetracycline, erythromycin, and sulfonamide. We employed an a priori sample size calculation based on our previous farmer market study [23]. We assumed a within-group standard deviation of 47.80 ppb and mean tetracycline residues of 50.00 ppb in beef, 31.31 ppb in eggs, and 92.00 ppb in honey. Considering unequal sample sizes and a Bonferroni correction for multiple comparisons, the required sample sizes for the food product groups were beef (*n*1) = 12, egg (*n*2) = 24, and honey (*n*3) = 12 (total sample size of 48). This sample size allowed us to achieve a power of 80% and a level of significance of 5% (two-sided) to detect statistically significant differences between the means of the three independent food product groups (beef, egg, and honey). Four grocery stores were selected purposively based on a couple of factors. Firstly, these grocery stores were locally popular and had high numbers of customer visits, making them representative of Knoxville’s overall consumer buying habits. Secondly, the stores were selected for easy access from the research laboratory at the College of Veterinary Medicine at the University of Tennessee, Knoxville. Beef, eggs, and honey were purchased from the selected grocery stores based on one criterion: the products could not contain any of the following on the label: “no antibiotic”, “no antimicrobials”, “antibiotic-free”, or “organic”. We selected these food animal products for our study based on two key criteria: frequent consumption by U.S. consumers and potential susceptibility to contamination with antimicrobial residues in the food chain. By focusing on commonly consumed food items with a high risk of antimicrobial residue contamination, our aim was to provide insights into the concentrations of antimicrobials in the food products sold as regular products at local grocery stores that are relevant to a large portion of U.S. consumers. Additionally, the food products (beef, eggs, and honey) were purchased from different production/brands/producers available at grocery stores on the sampling day. We visited four grocery stores and selected 12 eggs and 2–3 beef and honey from each grocery store, ensuring they came from different producers/brands/companies. We purchased the selected food products in their raw form. Subsequent to acquisition, the individual food products were allocated a distinctive code (ID) specifying the market origin, purchase date, and product classification. The beef and egg products were obtained in their conventional retail packaging. The honey was bought in its original sealed jar. Food products were delivered to the laboratory on ice within 2 h of collection. Beef products were maintained at −112 °F (−80 °C) in a freezer, whereas eggs were preserved under refrigerated conditions at temperatures ranging from 35.6 to 46.4 °F (2 to 8 °C). The honey products were maintained in a moisture-free environment, protected from direct solar exposure, at a controlled room temperature ranging from 68 to 77 °F (20 to 25 °C) until analysis [23]. To ensure the accuracy of our antimicrobial concentrations measurements using ELISA, we implemented several stringent contamination control measures. Dedicated equipment, including separate pipettes, tips, and containers, was used for each sample to prevent cross-contamination. Thorough cleaning protocols were followed, and all equipment and work surfaces were disinfected before and after each use. Additionally, reagents and sample processing occurred in a laminar flow hood to minimize exposure to contaminants. Food products were either analyzed immediately or stored at −112 °F (−80 °C), depending on the analysis timeline, to preserve their integrity. The preparation and processing of beef, egg, and honey products for tetracycline, erythromycin, and sulfonamide followed previously described methods [23].

### 2.2. Effect of Cooking Temperature on the Concentrations of Antimicrobials Spiked into Food Products

Samples in each group of food animal products (beef, eggs, and honey) were spiked with known concentrations of tetracycline, erythromycin, and sulfonamides (50, 100, and 150 µg/kg). The concentrations of spiked antimicrobial were chosen based on the literature [33,34,35]. Each food product was tested in triplicate for the control group (without cooking temperature treatment) and the treatment group (with cooking temperature). This resulted in a total of 504 individual samples analyzed (252 per group). Prior to spiking, all the food products were tested for the presence of tetracycline, erythromycin, and sulfonamide concentrations using the ELISA testing method, as previously described in the current study. Additionally, we used a blank sample (without antimicrobial spiking) as an experimental control. Each antimicrobial-spiked food sample was cooked for up to 30 min [18] until the USDA-recommended cooking temperature of 160 °F (71 °C) [36] was reached. After heat treatment, the samples were allowed to cool [18]. The preparation of samples for measuring antimicrobial concentrations by ELISA followed previously described methods [23].

### 2.3. Competitive ELISA Steps

We performed cELISA using a tetracycline ELISA Kit from Biovision to measure tetracycline concentrations, following the provided protocol (Biovision, CA, USA). Fifty microliters (μL) of the prepared sample was pipetted into each well of an ELISA plate precoated with an antibody specific to the target analyte. An additional 50 μL of antibody working solution was added to each well. The plate was then covered and incubated at 98.6 °F (37 °C) for 30 min in the dark. After incubation, the wells were emptied, and the plate was washed five times with 1X wash solution. Subsequently, 100 μL of enzyme conjugate was added to each well, followed by another 30 min of incubation at 98.6 °F (37 °C). The plate was washed again, and 50 μL each of the Substrate A and Substrate B solutions was added to the wells, followed by gentle shaking for 5 s. The plate was incubated at 98.6 °F (37 °C) for 15 min. After incubation, 50 μL of stop solution was added to each well and gently oscillated to halt the reaction. The optical density at 450 nm was then measured using a spectrophotometer within 10 min. The same protocol was followed for the cELISA assays for sulfonamide and erythromycin residues using their respective kits. Standard antimicrobial concentrations were calculated through nonlinear curve fitting with a Sigmoidal 4PL model using GraphPad Prism (version 9.5.1, La Jolla, San Diego, CA, USA).

### 2.4. Statistical Analysis

This study focused on tetracycline, erythromycin, and sulfonamide residue concentrations as the target antimicrobials. These concentrations were treated as three distinct continuous outcome variables. Descriptive and bivariate analyses were conducted as previously detailed [23]. Briefly, the Kruskal–Wallis test was used to assess statistically significant differences in median tetracycline concentrations. Since the Kruskal–Wallis test indicated significant differences, post hoc pairwise comparisons were conducted using Dunn’s test with Bonferroni correction to adjust for multiple comparisons [37]. In addition, the Mann–Whitney test was applied to compare mean erythromycin concentrations between beef and honey. Statistical significance was determined at a critical threshold of *p* < 0.05. Linear regression was used to analyze whether standard cooking to 160 °F (71 °C) reduced concentrations of antimicrobials (tetracycline, erythromycin, and sulfonamide) spiked into evaluated food products (beef, eggs, and honey). Separate linear regression models were conducted for each food type and stratified by spiking concentration. In these models, antimicrobial concentrations (µg/kg) served as the outcome variable, while the treatment condition (cooking vs. no cooking) was the primary exposure variable. The regression outcomes are presented as coefficients with 95% confidence intervals at each spiking concentration level. Statistical significance was determined at a *p*-value threshold of <0.05. Analyses were performed using Stata 17.0 [38] and GraphPad Prism 9 (La Jolla, CA, USA).

## 3. Results

### 3.1. Antimicrobial Concentrations in the Food Animal Products

A total of 65 food products were purchased. Tetracycline residues were detected in 100% of the beef (8/8), 96% of the eggs (46/48), and 100% of the honey (9/9). The median concentrations of the tetracycline residues were 7.73 µg/kg in beef, 5.62 µg/kg in eggs, and 13.13 µg/kg in honey (Table 1). None of the beef or egg products exceeded the FDA’s maximum residue limit (MRL) for tetracycline (Table 1). MRL is used throughout this manuscript to represent the USA’s tolerance limits. The Kruskal–Wallis test revealed a statistically significant difference in the median tetracycline concentrations across the three food types (*p* = 0.0002, Table 1). Pairwise comparisons showed no statistically significant differences in the residue concentrations between beef and eggs (*p* = 0.0516) or beef and honey (*p* = 0.3450). However, honey had a significantly higher concentration of tetracycline residues compared to eggs (*p* = 0.002).

Detectable erythromycin concentrations were found in the beef (5/8, 63%) and honey (9/9, 100%) products. The median erythromycin concentration was 0.14 µg/kg in beef and 0.48 µg/kg in honey. These values for all beef products were below the FDA’s MRL for erythromycin (Table 1). According to the Mann–Whitney test, there was no statistically significant difference I then erythromycin concentrations between beef and honey (*p* = 0.3853).

Regarding sulfonamide, all beef and egg products had no detectable sulfonamide concentrations (below the limit of detection of the ELISA kit) (Table 1).

### 3.2. Effect of Cooking Temperature on the Concentration of Antimicrobials Spiked into Food Products

The concentrations of tetracycline, erythromycin, and sulfonamide spiked into food products did not change significantly in response to cooking to 160 °F (71 °C). This result was consistent from 50 to 150 ug/kg for each antimicrobial drug in all tested tissue types.

## 4. Discussion

To the best of our knowledge, this study is the first to measure the concentrations of tetracycline, erythromycin, and sulfonamide in beef, eggs, and honey sold in conventional grocery stores in Knoxville, TN.

### 4.1. Antimicrobial Concentrations in Food Products

The study results indicated the presence of tetracycline and erythromycin residues at varying concentrations in beef, eggs, and honey obtained from conventional grocery stores. The detected antimicrobial concentrations in the beef and egg products were below the FDA’s established MRLs. No detectable sulfonamide concentrations were found in any beef or egg products obtained from the selected conventional grocery stores in Knoxville. Additionally, the study results indicate that the concentrations of tetracycline, erythromycin, and sulfonamide spiked into food products did not change significantly in response to the cooking temperature.

The concentrations of tetracycline, erythromycin, and sulfonamides observed in the current study were all below the FDA’s MRL, which is indicative that the foods were safe for consumption. The reasons for these antimicrobial concentrations could be that farmers adhered to the withdrawal periods when these animals are treated with selective antimicrobials. Alternatively, these animals may not have been directly treated with antimicrobials but were indirectly exposed to these antimicrobials from the environment. In support of this latter claim, a similar observation was made from an exploration of similar food products from farmers’ markets [23]. In that study [23], nonviolative concentrations of tetracycline, erythromycin, and sulfonamides were equally found in similar food products raised without the use of antimicrobials.

A review study reported that antimicrobial residues are frequently detected in various environments, including surface and drinking waters, sewage effluents, soils, sediments, and different plant species. These findings have been reported in countries such as China, Japan, South Korea, Europe, the USA, Canada, and India [39]. Additionally, Feng et al. (2020) reported the antimicrobial residues in drinking water sources in China [40]. Based on the relatively lower concentrations of antimicrobials found in the current study of foods from conventional grocery stores compared to those from farmers’ markets [23], it could be said that conventional foods may not be as bad as consumers think. They could be safer based on the antimicrobial concentrations found.

The current study found that the antimicrobial concentrations were lower in the honey products sold at conventional grocery stores than those sold at farmers’ markets [23]. Possible explanations for the presence of these antimicrobial concentrations include indirect exposure from the environment. Antimicrobial residues may contaminate honey when honeybees forage for or collect contaminated nectar and pollen. Additionally, honeybees might be exposed to water that contains antimicrobial residues. However, based on the relatively lower concentrations of antimicrobials detected in the current study in honey from grocery stores compared to those from farmers’ markets [23], it could be said that honey from conventional grocery stores could be safer in terms of antimicrobial concentrations. The U.S. FDA has not yet established MRLs for tetracycline and erythromycin residues in honey products. It is advisable to establish MRLs for tetracycline and erythromycin in the U.S.

While this study’s findings provide reassurance regarding the immediate safety of the examined food products, it is important to emphasize that while these antimicrobial concentrations are deemed safe and fall below the MRLs, the potential long-term effects of chronic exposure to low concentrations of antimicrobials remain a subject of concern. The cumulative impact of consuming low concentrations of antimicrobials over extended periods is not fully understood and warrants further investigation. Some researchers have suggested that even low-concentration exposure to antimicrobials could contribute to the development of AMR [41,42], which may also disrupt the healthy gut microbiota [43]. Future longitudinal research is needed to fully understand the adverse effects of long-term exposure to low concentrations of these antimicrobials.

The current study found that sulfonamide concentrations were undetectable in food products sold at conventional grocery stores compared to those sold at farmers’ markets [23], suggesting that foods from conventional grocery stores are safe for consumption and may contribute to reducing the exposure to sulfonamide among consumers. Possible reasons for the undetectable sulfonamide concentrations observed in grocery store products compared to farmers’ market food products [23] can be attributed to the contrasting farm management practices employed by large-scale commercial farms/operations supplying grocery stores and small-scale backyard farms/producers supplying farmers’ markets. Large-scale commercial farms typically implement standardized agricultural practices and maintain more rigorously controlled environments for their food animals. These operations often have better access to veterinary services/care and implement disease prevention protocols. Moreover, they may maintain meticulous records of antimicrobial administration, facilitating the more precise management of withdrawal periods before slaughter or the collection of eggs and honey. As a result, these farm management practices may reduce the necessity for antimicrobial use compared to that of smaller or backyard farms/producers that supply farmers’ markets.

### 4.2. Impact of Cooking Temperature on the Concentration of Antimicrobials Spiked into Food Products

The current study results indicated that cooking food products to 160 °F (71 °C) did not significantly change the concentrations of tetracycline, erythromycin, and sulfonamide that were spiked into the food products. This finding remained consistent across all tested food product types, with the spiked antimicrobial drug concentrations ranging from 50 to 150 µg/kg. This aligns with previous research suggesting the stability of tetracycline during cooking [25,44]. Gajda et al. reported only a 29.8% reduction in the doxycycline (a tetracycline) concentration in eggs, even after boiling [45]. Similarly, Canton et al. found that conventional cooking methods cannot eliminate all veterinary drugs in eggs [46]. Several factors could explain why cooking temperature had a limited effect in our study. The physical and chemical properties of the food itself, along with food additives, can influence how effectively heat degrades antimicrobials [47,48]. Fat content, pH, and potentially other unanalyzed factors in food products can influence the stability of antimicrobials [48,49,50,51]. A study indicated that the stability and degradation of tetracycline in food products are significantly influenced by the pH of the food [52]. For instance, tetracycline showed increased degradation at higher temperatures in neutral solutions compared to acidic solutions [50]. Furthermore, the presence of fat as a matrix component could impede the decrease in tetracycline concentrations following thermal treatment [51]. According to an experimental study that utilized the high-performance liquid chromatography (HPLC) method, tetracycline was found to degrade when exposed to both high humidity and temperature [53]. Additionally, a study has shown that tetracycline degrades after being exposed to ultraviolet light [53]. Future laboratory-based experimental research utilizing the HPLC method could be employed to evaluate the degradation of antimicrobials across different food animal products (beef, egg, and honey) at different cooking temperatures to gain valuable insights. It would also be important to explore the potency or antimicrobial activity at these concentrations following cooking in future studies.

### 4.3. Study Limitations and Future Research Directions

The present study focused on whether the FDA-recommended cooking temperature of 160 °F (71 °C) for inactivating the most common pathogenic bacteria in foods influenced the spiked drug concentration in food products and did not determine whether varying cooking temperature reduced the concentrations of the antimicrobials spiked into the evaluated food products. Furthermore, this investigation utilized different concentrations of antimicrobials spiked into food products under controlled conditions to simulate the behavior of naturally occurring residues in food tissue. This may not have fully replicated the distribution or behavior of the residues naturally occurring in food products. Also, it is important to note that antimicrobial drug concentrations may be distributed differently at the subcellular level under natural conditions, which could influence the magnitude of the effect of cooking temperature on concentrations of antimicrobials in food products. Future research could investigate the behavior of naturally occurring antimicrobial residues under different cooking conditions to complement the findings of this study.

In this study, ELISA was utilized to quantify the concentrations of tetracycline, erythromycin, and sulfonamide residue in food animal products. This method is simple, fast, and cost-effective compared to other methods, such as high-performance liquid chromatography (HPLC), liquid chromatography-mass spectrometry (LC-MS), or mass spectrometry (MS) [54]. However, it may not be as sensitive as these other methods. Additionally, spectrophotometry, commonly used for ELISA plate readings in our laboratories, has limitations in sensitivity [55] and can be affected by food matrix components [56]. Due to budgetary and logistical constraints, we did not use HPLC to confirm cELISA-positive food samples. Nevertheless, a previous study has shown a significant correlation (r = 0.93, *p* < 0.01) between HPLC and cELISA for detecting tetracycline residues in food animal products [54]. Also, there is a correlation between HPLC and cELISA in measuring sulfonamide residues (r = 0.60, *p* < 0.05) in food products [54]. We did not find any relevant published article that reported a correlation between HPLC and cELISA in measuring erythromycin residues (macrolide). However, there is evidence of a significant correlation between liquid chromatography-mass spectrometry (LC/MS) and ELSA in determining tylosin (macrolide) residues (r = 0.93, *p* < 0.001) in pig plasma. ELISA is a simple and low-cost method for the quantitative determination of antimicrobial concentrations in food and animal products [57,58,59]. Due to resource constraints, we could not assess the thermal stability or degradation of tetracycline in the three food groups at different cooking temperatures using both the ELISA and HPLC methods. Future research should be conducted in a laboratory setting to evaluate the thermal stability and degradation of tetracycline and identify the specific factors responsible for the stability across different food groups. This research should involve comparing the ELISA with the HPLC method to obtain more comprehensive information on tetracycline’s thermal stability and degradation at various cooking temperatures across different food groups.

## 5. Conclusions

This study revealed that the median or mean concentrations of tetracycline, erythromycin, and sulfonamide residues in beef and eggs from conventional grocery stores were within safe/allowable limits compared to MRLs. Since MRLs have not been established for erythromycin and tetracycline in honey products in the U.S., we cannot infer if the residue concentrations fall within safe/allowable limits. Furthermore, the cooking temperature did not significantly reduce the concentrations of the tetracycline, erythromycin, and sulfonamide spiked into the food products in this study. Further research is necessary to examine the contributing factors related to tetracycline and erythromycin residues in food products sold at conventional grocery stores. This will help to identify any controllable factors for implementing effective control measures.

## Figures and Tables

**Table 1:** Tetracycline, erythromycin, and sulfonamide concentrations in food animal products sold at conventional grocery stores in Knoxville (USA) during summer 2021.

Type of Sample	Total Samples	x/n	Antimicrobial Concentrations (µg/kg)	MRL ^1^ (µg/kg)	MRL ^2^ (µg/kg)	Exceed MRL (U.S. FDA) n (%)	*p*-Value
			Tetracycline				
			Median (IQR)				
Beef	8	8/8	7.73 (6.86, 12.08)	200	100	0 (0)	
Egg	48	46/48	5.62 (4.20, 7.52)	200	200	0 (0)	0.0002 ^a^
Honey	9	9/9	13.13 (10.88, 13.97)	NA	NA	NA	
			Erythromycin				
			Median (IQR)				
Beef	8	5/8	0.14 (0, 3.19)	100	200		0.3853 ^b^
Honey	9	9/9	0.48 (0.34, 0.64)	NA	NA		
			Sulfonamides				
Beef	8	0/8	0	100	100	0 (0)	
Egg	48	0/48	0	100	NA	0 (0)	

Footnotes: SD = standard deviation. MRL = maximum residue limits. NA = not applicable. IQR = interquartile range. x, number of samples had detectable antimicrobial concentrations. n, number of samples tested. ^1^ MRL set by the U.S. FDA. ^2^ MRL set by the European Union. ^a^ *p* values were obtained from the Kruskal–Wallis test. ^b^ *p*-values were obtained from the Mann–Whitney test. For tetracycline, erythromycin, and sulfonamide, “0” means below the limit of detection.

## Data Availability

The original contributions presented in this study are included in this article Further inquiries can be directed to the corresponding author.
